# Respiratory Muscle Function Tests and Diaphragm Ultrasound Predict Nocturnal Hypoventilation in Slowly Progressive Myopathies

**DOI:** 10.3389/fneur.2021.731865

**Published:** 2021-10-14

**Authors:** Jens Spiesshoefer, Riccarda Lutter, Hans-Joachim Kabitz, Carolin Henke, Simon Herkenrath, Winfried Randerath, Peter Young, Michael Dreher, Dennis Görlich, Matthias Boentert

**Affiliations:** ^1^Department of Neurology With Institute for Translational Neurology, Muenster University Hospital, Muenster, Germany; ^2^Department of Pneumology and Intensive Care Medicine, Aachen University Hospital, Aachen, Germany; ^3^Department of Pneumology, Cardiology, and Intensive Care Medicine, Klinikum Konstanz, Konstanz, Germany; ^4^Department of Neurology, Herz-Jesu-Krankenhaus Münster-Hiltrup, Münster, Germany; ^5^Bethanien Hospital gGmbH Solingen, Solingen, Germany; ^6^Institute for Pneumology, University of Cologne, Solingen, Germany; ^7^Medical Park Klinik Reithofpark, Bad Feilnbach, Germany; ^8^Institute of Biostatistics and Clinical Research, University of Muenster, Muenster, Germany; ^9^Department of Medicine, Universitätsklinikum Münster (UKM) Marienhospital, Steinfurt, Germany

**Keywords:** myopathy, diaphragm ultrasound, maximum inspiratory pressure, nocturnal hypoventilation, forced vital capacity

## Abstract

**Introduction:** In slowly progressive myopathies, diaphragm weakness early manifests through sleep-related hypoventilation as reflected by nocturnal hypercapnia. This study investigated whether daytime tests of respiratory muscle function and diaphragm ultrasound predict hypercapnia during sleep.

**Methods:** Twenty-seven patients with genetic myopathies (myotonic dystrophy type 1 and 2, late-onset Pompe disease, facioscapulohumeral dystrophy; 48 ± 11 years) underwent overnight transcutaneous capnometry, spirometry, measurement of mouth occlusion pressures, and diaphragm ultrasound.

**Results:** Sixteen out of 27 patients showed nocturnal hypercapnia (peak p_tc_CO_2_ ≥ 50 mmHg for ≥ 30 min or increase in p_tc_CO_2_ by 10 mmHg or more from the baseline value). In these patients, forced vital capacity (FVC; % predicted) and maximum inspiratory pressure (MIP; % of lower limit or normal or LLN) were significantly reduced compared to normocapnic individuals. Nocturnal hypercapnia was predicted by reduction in FVC of <60% [sensitivity, 1.0; area under the curve (AUC), 0.82] and MIP (%LLN) <120% (sensitivity, 0.83; AUC, 0.84), the latter reflecting that in patients with neuromuscular disease, pretest likelihood of abnormality is *per se* higher than in healthy subjects. Diaphragm excursion velocity during a sniff maneuver excluded nocturnal hypercapnia with high sensitivity (0.90) using a cutoff of 8.0 cm/s.

**Conclusion:** In slowly progressive myopathies, nocturnal hypercapnia is predicted by FVC <60% or MIP <120% (LLN). As a novelty, nocturnal hypercapnia can be excluded with acceptable sensitivity by diaphragm excursion velocity >8.0 cm/s on diaphragm ultrasound.

## Introduction

In patients with neuromuscular disorders, respiratory muscle involvement is common and a major cause of morbidity and mortality (1). Whereas overall prognosis is most affected in amyotrophic lateral sclerosis and Duchenne's muscular dystrophy (DMD) (2, 3), respiratory muscle weakness may also evolve in slowly progressive conditions, including hereditary myopathies such as late-onset Pompe disease or myotonic dystrophy type 1 (4, 5). Pathophysiologically, respiratory muscle dysfunction leads to alveolar hypoventilation and retention of carbon dioxide (CO_2_), which usually manifests during rapid eye movement sleep first (6). With disease progression, nocturnal hypercapnia may spread to non-rapid eye movement (non-REM) sleep stages, eventually followed by daytime hypercapnia and type II respiratory failure. Regarding diagnostic sleep studies, transcutaneous capnometry has been shown to be superior to pulse oxymetry for detection of sleep-related hypoventilation (7, 8). Since capnometry is still not widely available in many countries, objective daytime predictors of nocturnal hypercapnia are desirable in order to early identify patients who should be transferred to specialized sleep centers for further evaluation of sleep-related breathing and, if indicated, start of nocturnal non-invasive ventilation (NIV). Although the cumulative prevalence of progressive neuromuscular disorders exceeds 50 per 100,000 (9), only few studies specifically investigated predictors of sleep-related hypoventilation in this population (10–12). In juvenile patients with DMD, forced expiratory volume in 1 s below 40% predicted was reported to predict hypoventilation during sleep (10, 11), and in a mixed cohort of adult DMD and non-DMD patients, one study elegantly showed that inspiratory vital capacity and maximum inspiratory pressure (MIP) both predict and quantitatively reflect hypercapnia during sleep or at daytime, respectively (12). Since comparable evidence is still missing for patients with slowly progressive myopathies and muscular dystrophies the present study evaluated whether daytime tests of respiratory muscle strength and function predict nocturnal hypercapnia in this population. Furthermore, this study supplemented bedside diagnostic tests of respiratory muscle function by diaphragm ultrasound. The latter has been established as an assessment tool for inspiratory muscle strength and function that is both non-invasive and widely available (13).

## Patients and Methods

### Experimental Study Design

This cross-sectional study was conducted from November 2017 to March 2019. Ethical approval was obtained from the local ethics committee (Ethikkommission der Ärztekammer Westfalen-Lippe und der WWU Münster, Az. 2016-072-f-S). All participants gave their written informed consent to participate in this study.

### Study Population

Patients with genetically proven slowly progressive myopathies were consecutively recruited from an academic neuromuscular specialty clinic. Diagnoses included myotonic dystrophy type 1 and type 2 (DM1 and DM2), facioscapulohumeral muscular dystrophy type 1 (FSHD1), and late-onset Pompe disease. In these conditions, respiratory muscle involvement and sleep-disordered breathing have been previously described (4, 14–16).

This study was part of a wider project investigating the pathophysiology of respiratory muscle strength and function in neuromuscular disorders and chronic obstructive pulmonary disease (ClinicalTrials.gov Identifier: NCT03032562).

### Clinical Assessment

Apart from demographic and anthropometric data, clinical information on the individual neurological status was collected. Motor function of both arms and legs was categorized according to the Brooke and Vignos scales, which have originally been introduced for functional assessment of DMD patients. The Brooke scale ranges from “1” (“Can abduct arms in full circle until they touch above head”) to “6” (“Cannot raise hands to mouth and has no useful hand function”) (17). The Vignos scale ranges from “1” (“Walks and climbs stairs without assistance”) to “10” (“Confined to bed”) (18).

### Spirometry, Maximum Inspiratory, and Expiratory Pressures

Lung function tests were performed according to standard recommendations using an electronic spirometer (Vitalograph 3000™, Vitalograph, Hamburg, Germany) (19). Participants performed a maximum effort toward their individual forced vital capacity (FVC) and forced expiratory volume in the first second (FEV1) in the upright sitting position. Results of at least five consecutive attempts were collected until the highest value was achieved and showed <10% variation from the preceding test. FVC and FEV1 were expressed as percentage of the predicted value based on gender, height, and age. Reference values were derived from the 2012 Global Lung Initiative database (20). Maximum inspiratory pressure (MIP) was obtained using a handheld electronic manometer (MicroRPM™, Care Fusion, Baesweiler, Germany), and test standardization and analysis were in accordance with current guidelines (19). Predicted values and lower limits of normal (LLN) for MIP and MEP were calculated as proposed by Evans and Whitelaw (21). The peak cough flow (PCF) was measured using a standard peak flow meter (19). For all measurements, a nasal clip was used to prevent air leakage.

### Diaphragm Ultrasound

**Figure 1:**
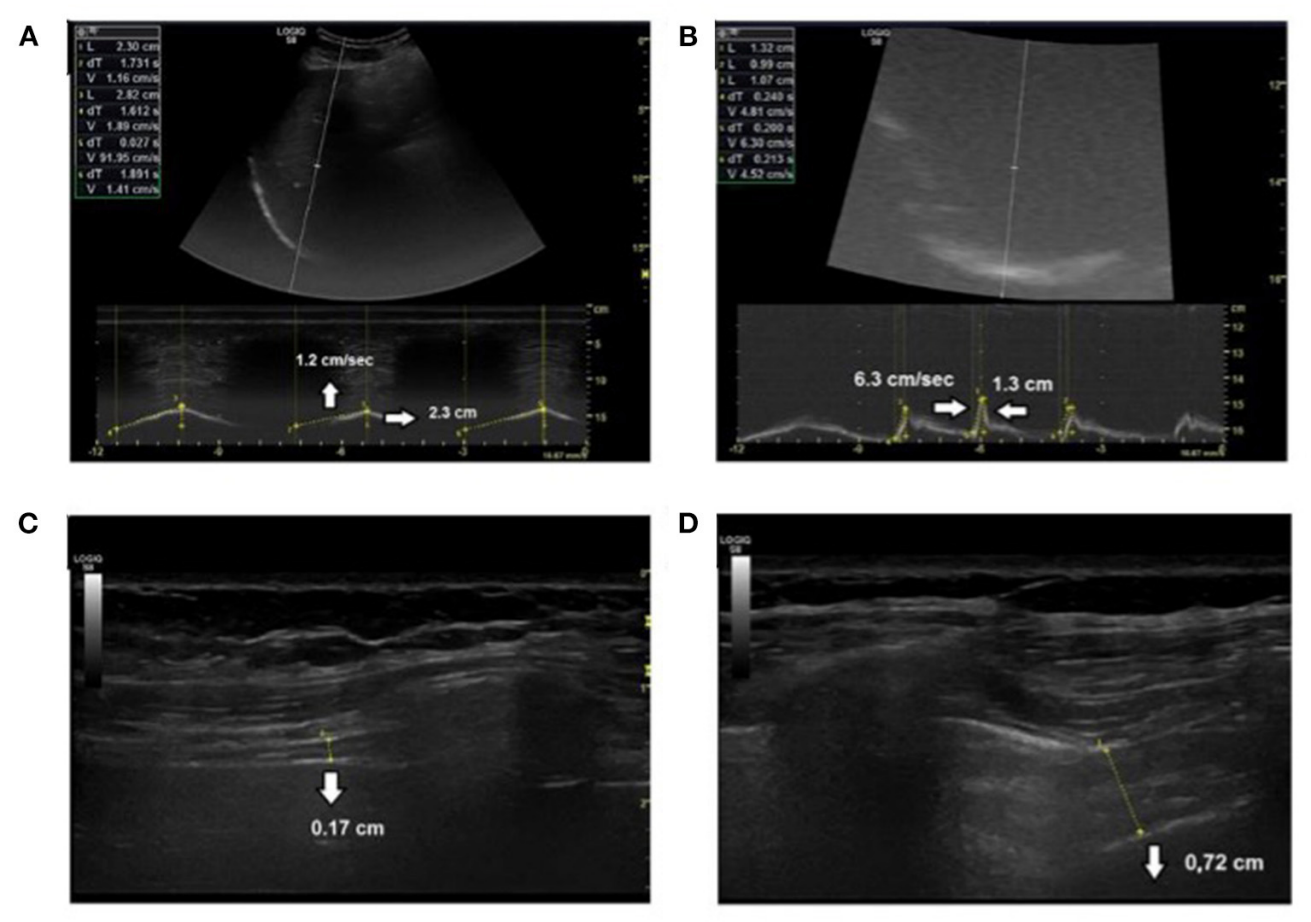
Diaphragm ultrasound measures. **(A)** Diaphragm excursion amplitude and velocity during tidal breathing, **(B)** diaphragm excursion amplitude and velocity during voluntary sniff, **(C)** diaphragm thickness at functional residual capacity, and **(D)** at total lung capacity.

Diaphragm ultrasound was performed by one experienced investigator (JS) who applied a standardized protocol for examination of the right hemidiaphragm in the supine position as previously described (22). A portable ultrasound machine (LOGIQ S8-XD clear™, GE Healthcare, London, UK) with a 3.5-MHz convex transducer was used for assessment of diaphragm excursions. The probe was positioned subcostally between the mid-clavicular and anterior axillary lines. Diaphragm excursion amplitude was measured as the range of diaphragm displacement during tidal breathing, after maximum inspiration, and following a voluntary sniff maneuver (Figure 1). Diaphragm excursion velocity was assessed during tidal breathing and following a maximum sniff only. A 10-MHz linear transducer was used for assessment of diaphragm thickness in the zone of apposition. Diaphragm thickness (defined as the distance between the inner part of the pleural layer and the inner part of the peritoneal layer) was measured at both functional residual capacity (FRC) and total lung capacity (TLC). The probe was positioned in the posterior axillary line between the 8th and 10th intercostal space. Diaphragm thickening ratio was calculated as thickness at TLC divided by thickness at FRC. All measurements were performed thrice at least after careful instruction of the patient, and maximum values were taken for statistical analysis.

### Sleep Studies

Diagnostic sleep studies comprised cardiorespiratory polygraphy (Weinmann, Hamburg, Germany) or polysomnography (Nihon Kohden, Rosbach, Germany), which was performed and evaluated according to standard recommendations (23). We recorded respiratory parameters including the peripheral oxygen saturation (SpO_2_). Transcutaneous capnometry (Sentec, Therwil, Switzerland) was performed along with each polygraphy or polysomnography, respectively (24). Nighttime hypercapnia was diagnosed when peak transcutaneous carbon dioxide tension (p_tc_CO_2_) was ≥50 mmHg for 30 min at least, or when nocturnal p_tc_CO_2_ increased from the awake baseline value by 10 mmHg or more (25). Early morning capillary blood gases were drawn from the arterialized earlobe, and daytime hypercapnia was defined by a pCO_2_ ≥45 mmHg (25).

### Statistical Analysis

All analyses were performed using SPSS® 24.0 (IBM Inc., Armonk, NY, USA). Results are expressed as mean and standard deviation for continuous variables with normal distribution, and median and interquartile range for continuous variables with a skewed distribution. Categorical variables are expressed as percentages, unless otherwise specified. Differences between groups were analyzed using the unpaired *T*-test or the Mann–Whitney rank sum test, while differences in categorical data were compared using the χ^2^-test. Diagnostic ability of different cutoff values for FVC, MIP, and ultrasound measures to predict nocturnal hypercapnia was tested by means of receiver-operating characteristics (ROC) analysis. Sensitivity, specificity, and the Youden index (specificity + sensitivity – 1) were determined for each value. The maximum Youden index was used to select the most appropriate cutoff score. Intercorrelation of continuous variables was performed using Spearman's correlation coefficient, and Bonferroni's correction was applied for multiple correlations. For all analyses, a *p* <0.05 was considered statistically significant. For graphical illustrations GraphPad Prism™ version 7 (Graphpad Software, San Diego, CA) was used.

## Results

### Patients and Sleep-Related Breathing

**Figure 2:**
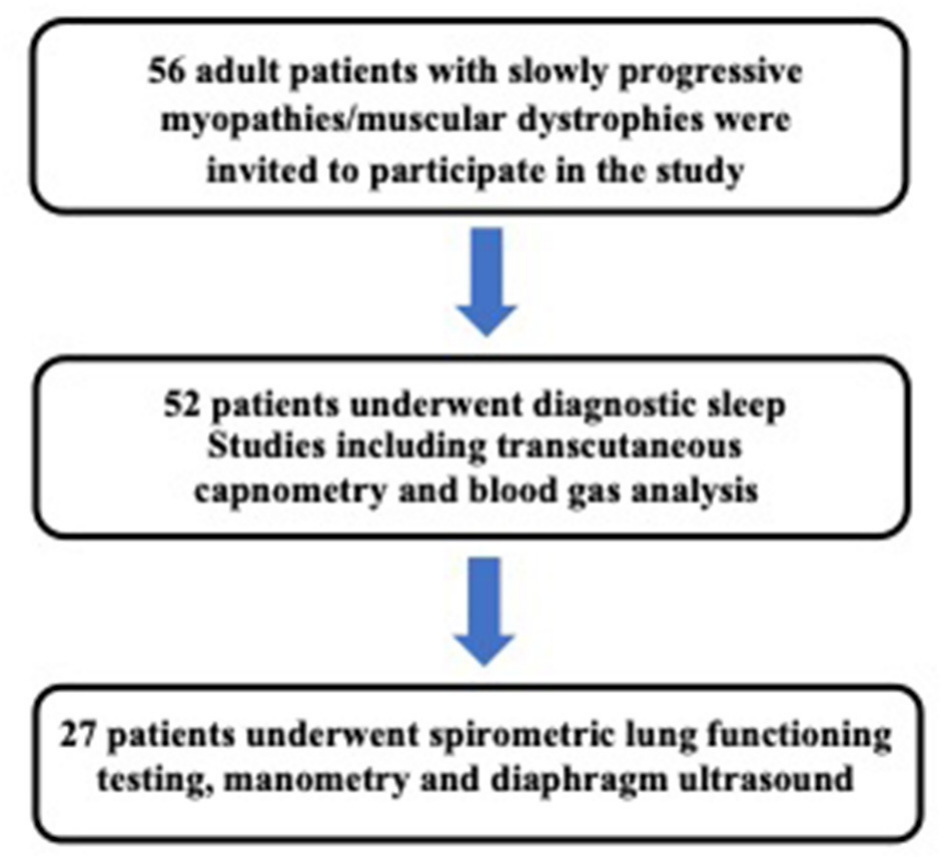
Study flow chart and methodology.

Table 1 summarizes demographic and anthropometric characteristics of the study participants, and Figure 2 presents a study flow chart. Finally, 27 patients [age 48 ± 11 years; 67% male; body mass index (BMI), 26 ± 5 kg/m^2^] were enrolled in the study. Thirteen patients were diagnosed with DM1, one with DM2, five with late-onset Pompe disease, and eight patients with FSHD1. One patient with FSHD1 was wheelchair bound. No patient showed significant kyphoscoliosis. In 16 patients, nighttime NIV had previously been established in our academic sleep laboratory based on the presence of nocturnal hypoventilation. Treatment adherence to NIV was highly variable among this group, and none of the patients used NIV for more than 12 h, per 24-h day. Diagnostic sleep studies (i.e., without NIV use) including transcutaneous capnometry were available in all patients. Daytime hypercapnia (as defined by pCO_2_ ≥45 mmHg on blood gas analysis) was not found in any of the patients enrolled. Nocturnal hypercapnia as defined by nocturnal peak p_tc_CO_2_ ≥50 mmHg for ≥30 min or an increase in p_tc_CO_2_ above the awake baseline by ≥10 mmHg was present in the 16 NIV users only. This group comprised eight patients with DM1, four patients with Pompe disease, and four patients with FSHD. In these individuals, daytime pCO_2_ was significantly higher than in the 11 patients without nocturnal hypercapnia (41.0 ± 3.9 mmHg vs. 35.5 ± 2.6 mmHg, *p* = 0.001; Table 1). In the entire cohort, daytime pCO_2_ was associated with the maximum nocturnal p_tc_CO_2_ (*r* = 0.48, *p* = 0.023). In 19 of 27 patients, the apnea hypopnea index (AHI) was ≥5/h of recording time. Median AHI was 12.3/h with no significant difference between patients with and without nocturnal hypercapnia (data not shown). Median values for obstructive, central, and mixed apnea indices, and the hypopnea index were 3.8, 0.9, 0.1, and 4.5/h, respectively.

**Table 1:** Demographic, clinical, basic lung function data and blood gas analysis in patients with and without nocturnal hypoventilation.

	**No NH (*n* = 11)**	**NH (*n* = 16)**	***p*-value**
**Clinical data**			
Male, *n* (%)	5 (50.0)	14 (87.5)	n.s.
Age, years	50.9 ± 12.0	46.3 ± 17.2	n.s.
Body mass index, kg/m^2^	26.2 ± 4.4	25.6 ± 5.3	n.s.
Brooke scale score (arms)	1.4 ± 1.0	1.7 ± 1.0	n.s.
Vignos scale score (legs)	2.1 ± 1.6	2.5 ± 2.2	n.s.
**Lung function tests**			
FVC, L	3.1 ± 1.0	2.5 ± 1.2	n.s.
FVC, % predicted	76.8 ± 11.9	54.9 ± 19.5	**0.003**
FEV1, % predicted	72.7 ± 7.1	57.9 ± 18.0	**0.017**
FEV1/FVC, %	79.3 ± 8.6	75.2 ± 24.1	n. s.
PEF, L/sec	5.4 ± 1.3	6.0 ± 2.2	n. s.
PEF, % predicted	69.5 ± 11.2	66.9 ± 17.9	n. s.
PCF, L/min	292.7 ± 76.7	276.7 ± 89.2	n. s.
MIP, cmH_2_O	57.6 ± 26.5	38.4 ± 18.5	**0.03**
MIP, % predicted	64.6 ± 26.2	38.6 ± 15.8	**0.004**
MIP, % LLN	124.7 ± 48.5	72.9 ± 28.5	**0.002**
MEP, cmH_2_O	59.0 ± 40.5	56.3 ± 27.5	n. s.
MEP, % predicted	52.5 ± 29.5	49.2 ± 33.2	n. s.
MEP, % LLN	84.5 ± 60.7	80.3 ± 52.1	n. s
**Blood gas analysis**			
pH	7.44 ± 0.03	7.41 ± 0.02	**0.021**
pO_2_, mmHg	80.8 ± 12.7	80.1 ± 18.5	n.s.
pCO_2_, mmHg	35.5 ± 2.6	41.0 ± 3.9	**0.001**
SBC, mmol/l	26.0 ± 1.6	25.7 ± 1.6	n.s.
BE, mmol/l	0.73 ± 2.1	1.7 ± 2.1	n.s.
SpO_2_, %	96.3 ± 1.6	95.4 ± 3.1	n.s.

*Data are presented as mean and standard deviation, number of patients or percentage as indicated.*

*NH, nocturnal hypoventilation; n.s., not significant; FVC, forced vital capacity; FEV1, forced exspiratory volume in 1 s; PEF, peak expiratory flow; PCF, peak cough flow; MIP, maximum inspiratory pressure; MIP, % LLN, maximum inspiratory pressure as percentage of its lower limit of normal, MEP, maximum expiratory pressure; MEP,% LLN, maximum expiratory pressure as percentage of its lower limit of normal; pO_2_, oxygen partial pressure; pCO_2_, carbon dioxide partial pressure; SBC, standard bicarbonate; BE, base excess; SpO_2_, peripheral oxygen saturation.*

*P-values <0.05 are depicted as bold numbers*.

### Tests of Respiratory Muscle Function, Diaphragm Ultrasound, and Nocturnal Hypoventilation

In all study participants, respiratory muscle strength testing was performed along with diagnostic sleep studies (*n* = 17) or within 6 months at maximum (*n* = 10). In the latter group, no significant morbidity, hospitalization, or worsening of the neurological status occurred between the two testing dates. Among the entire study cohort, FVC, MIP, and MEP were all moderately reduced as compared to guideline-based reference values (FVC, 63.8 ± 19.8% predicted; MIP, 49.2 ± 24.1% predicted; MEP, 50.5 ± 31.2% predicted). Functional scores (Brooke and Vignos clinical scales) did not significantly differ between patients with and without nocturnal hypercapnia (data not shown).

**Figure 3:**
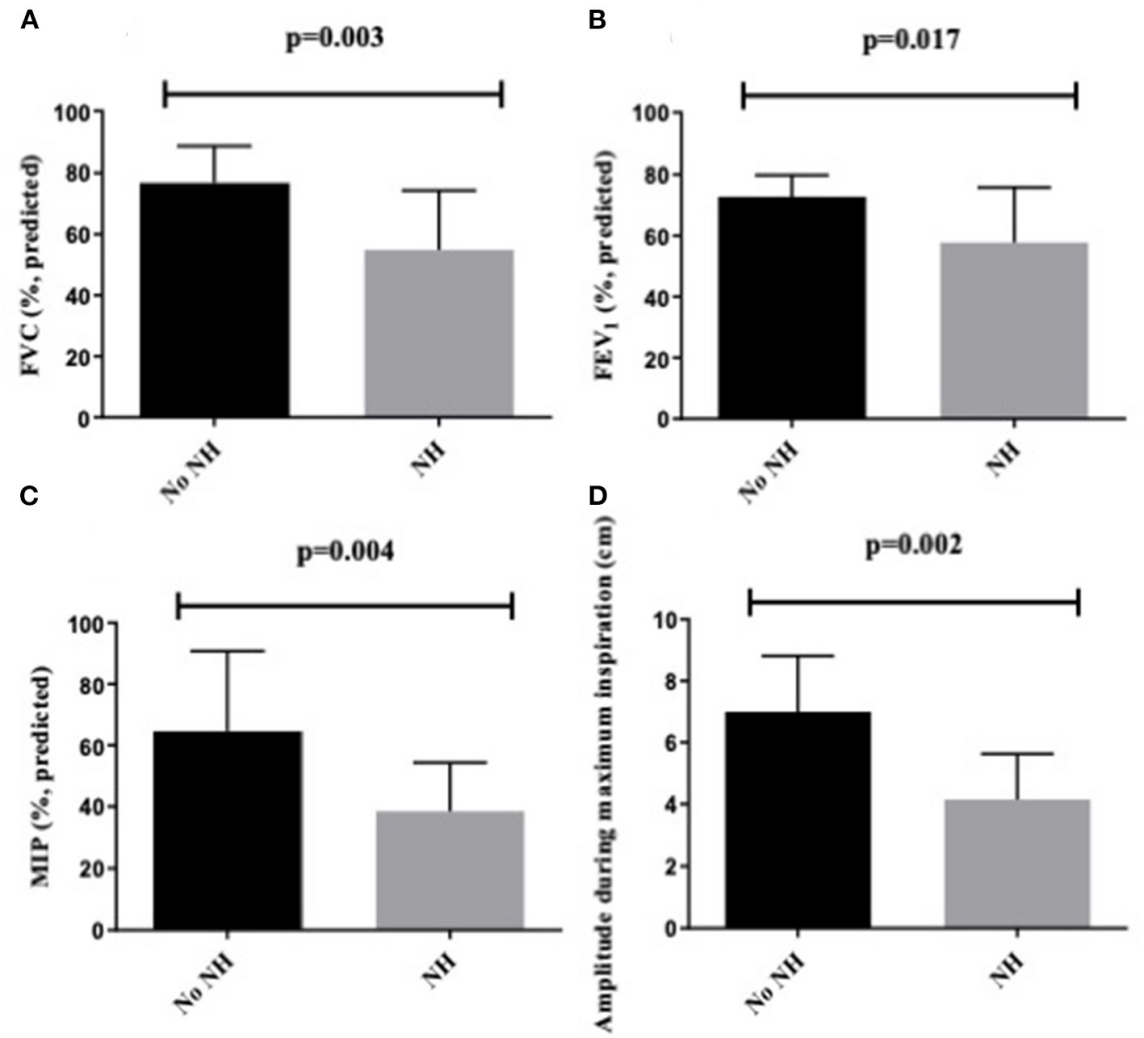
**(A)** FVC as % predicted, **(B)** FEV1 as % predicted, **(C)** MIP as % predicted, **(D)** diaphragm excursion amplitude during maximum inspiration in cm in patients with and without NH. NH, nocturnal hypoventilation; FVC, forced vital capacity; FEV1, forced expiratory volume in 1 s; MIP, maximum inspiratory pressure.

In patients with nocturnal hypercapnia, FVC, MIP, and FEV1 were significantly lower than in normocapnic individuals (Table 1; Figures 3A–C). ROC analysis revealed that nighttime hypercapnia could be predicted by FVC using a threshold of <60% predicted (area under the curve 0.82; *p* = 0.007; sensitivity, 1.0; positive predictive value, 1.0; specificity, 0.63). The same hold true for MIP <120% of the LLN (area under the curve, 0.84; *p* = 0.004; sensitivity 0.83; positive predictive value, 0.94; specificity, 0.73).

On diaphragm ultrasound, excursion amplitude during maximum inspiration, diaphragm thickness at TLC, and diaphragm thickening ratio were markedly reduced in all patients when compared to reference values previously published (22). Diaphragm excursion amplitude was significantly correlated with FVC (% predicted) and absolute MIP (Table 2). Significant correlations were also found between diaphragm thickening ratio and FVC and diaphragm thickness at TLC and FVC (% predicted; Table 2).

**Table 2:** Correlation analysis between diaphragm ultrasound parameters and bedside tests of respiratory muscle function.

	**FVC (% predicted)**	**MIP (cmH_**2**_O)**
Excursion amplitude	0.80 (*p* <0.001)	0.79 (*p* <0.001)
Thickness at FRC	n. s.	n. s.
Thickness at TLC	n. s.	0.73 (*p* <0.001)
DTR	0.66 (*p* <0.001)	n. s.
Sniff velocity	n. s.	n. s.

*The table depicts Spearman's correlation coefficients and p-values (in brackets). Correlation analysis was performed in the entire study cohort (n = 27).*

*DTR, diaphragm thickening ratio; FRC, functional residual capacity; FVC, forced vital capacity; MIP, maximum inspiratory pressure; TLC, total lung capacity*.

In patients with nocturnal hypercapnia diaphragm, excursion amplitude during maximum inspiration but not the diaphragm thickening ratio was significantly lower than in individuals with nighttime normocapnia (4.15 ± 1.48 vs. 7.00 ± 1.82 cm; *p* = 0.002) (Table 3; Figure 3D). ROC analysis did not prove diaphragm excursion amplitude during maximum inspiration or excursion amplitude during a voluntary sniff to be predictive for nighttime hypercapnia (data not shown). However, sensitivity of sniff velocity to exclude nocturnal hypercapnia was 90% using a cutoff of 8.0 cm/s (area under the curve, 0.73; *p* = 0.04).

**Table 3:** Diaphragm ultrasound parameters in patients with and without nocturnal hypoventilation.

	**Reference values (22)**	**No NH (*n* = 11)**	**NH (*n* = 16)**	***p*-value**
**Diaphragm excursion**				
Amplitude during tidal breathing, cm	1.56 ± 0.53	1.61 ± 0.50	1.68 ± 0.74	0.80
Velocity during tidal breathing, cm/s	1.12 ± 0.44	1.16 ± 0.35	1.29 ± 0.48	0.48
Amplitude during voluntary sniff, cm	2.52 ± 1.00	2.56 ± 0.96	1.89 ± 0.82	0.12
Velocity during voluntary sniff, cm/s	6.82 ± 2.03	6.60 ± 2.79	5.77 ± 2.81	0.53
Amplitude during max. inspiration, cm	8.02 ± 1.91	7.00 ± 1.82	4.15 ± 1.48	**0.002**
**Diaphragm thickness**				
at FRC, cm	0.19 ± 0.06	0.19 ± 0.09	0.17 ± 0.08	0.69
at TLC, cm	0.53 ± 0.18	0.37 ± 0.17	0.29 ± 0.12	0.16
DTR	2.86 ± 0.88	2.10 ± 0.55	1.79 ± 0.48	0.15

*Data are presented as mean ± standard deviation. In column 2, reference values are provided for healthy subjects irrespective of gender (22). Group comparison was carried out between patients with and without NH.*

*NH, nocturnal hypoventilation; n.s., not significant; FRC, functional residual capacity; TLC, total lung capacity; DTR, diaphragm thickening ratio.*

*P-values < 0.05 are depicted as bold numbers*.

## Discussion

The present study determined the diagnostic accuracy of daytime tests of respiratory muscle strength and function with regard to sleep-related hypoventilation as reflected by nocturnal hypercapnia in adult patients with slowly progressive myopathies. The bedside measures that were evaluated comprised spirometry, manometry, and diaphragm ultrasound. Nocturnal hypercapnia was defined as p_tc_CO_2_ ≥ 50 mmHg for ≥30 min or an overnight increase in the p_tc_CO_2_ of ≥10 mmHg. The main finding of this study is that in slowly progressive myopathies, reduction in FVC and MIP reliably predict nocturnal hypercapnia when specific thresholds are applied (<60% of predicted for FVC and <120% of LLN for MIP). In contrast, sniff velocity on diaphragm ultrasound can only exclude the presence of nighttime hypercapnia with acceptable sensitivity when it exceeds 8.0 cm/s.

Previous studies have shown that vital capacity as a global measure of lung and respiratory muscle function allows prediction of sleep-related hypoventilation in patients with neuromuscular disorders (10–12). In a mixed cohort of children and adolescents with DMD, limb girdle muscular dystrophies, Pompe disease, and spinal muscular atrophy, Mellies et al. showed that nocturnal hypercapnia can be assumed when IVC falls below 40% of the predicted value (11). This finding could be confirmed in adult patients with progressive myopathies (12). Importantly, these studies used different temporal thresholds for definition of nocturnal hypercapnia [either p_tc_CO_2_ >50 mmHg for 50% of total sleep time (11) or p_tc_CO_2_ >50 mmHg for >50% of REM sleep alone or during both REM and >50% of non-REM sleep (12)]. The latter study revealed that intermittent CO_2_ retention during REM sleep can be predicted by IVC <60%, and continuous hypercapnia during sleep can be expected if IVC falls below 40% (12). Reduction in MIP was also found to be a strong predictor of nocturnal hypercapnia (12). The present study could show that reduction in FVC below 60% of the predicted value indicates nocturnal hypercapnia also in slowly progressive myopathies. It has to be taken into account that FVC and MIP testing may be hampered by weakness of mouth closure, which is present in many patients with neuromuscular disorders. To circumvent this problem, the sniff nasal inspiratory pressure (SNIP) has been reported to predict indication for NIV in patients with amyotrophic lateral sclerosis, for example (26). In slowly progressive myopathies, this test has not yet been studied in conjunction with sleep-related breathing. This holds also true for the present study in which mouth leakage was either absent or could be prevented by using a face mask for MIP and FVC testing, if necessary. However, future studies should comprise measurement of both MIP and SNIP, since the sniff maneuver is considered more physiological than forced inspiration against an occluded airway. Bedside tests of lung function and respiratory muscle strength are volitional in nature and may show substantial intraindividual variation (26, 27). As a non-volitional test, invasive measurement of the transdiaphragmatic pressure following phrenic nerve stimulation is an established method but requires substantial technical effort and nasal insertion of balloon catheters. Diaphragm ultrasound has emerged as a tool to study diaphragm function, and it has been shown that diaphragm excursion velocity during a sniff maneuver and diaphragm thickening ratio may reflect inspiratory muscle function and, potentially, strength (13, 22, 28–30). However, diaphragm ultrasound still is a volitional method that depends on patients' cooperation and does not yield truly objective results. Furthermore, valid data acquisition requires specifically trained personnel and structured protocols for conducting diaphragm sonography as previously proposed (13, 22).

To the best of our knowledge, no study has yet combined ultrasound parameters, MIP and FVC, in the context of sleep-related hypoventilation in patients with neuromuscular disease. Regarding MIP and FVC, our findings confirm previous studies and show that both measures are suitable to predict or rule out sleep-related hypercapnia. Of note, it may be considered conflicting that in this study, MIP <120% LLN turned out to be predictive of nocturnal hypercapnia, i.e., including values above the calculated LLN. However, published reference values for predicted mean and LLN of the MIP show substantial variation, including inconsistent sensitivity with regard to the pretest likelihood of diaphragm weakness that is naturally increased in subjects with neuromuscular disease (31). Thus, it appears to be logically consistent that for patients with known or suspected diaphragm weakness the threshold of normality is higher than values that were obtained from healthy individuals. The present study underlines that assessment of MIP has to be embedded in the clinical context, and concordance between test interpretation and pretest probability of abnormality is required in order to guide clinical decision-making (i.e., whether sleep studies and overnight capnometry should be initiated in a given patient).

As a novelty, the present study shows that diaphragm excursion velocity during maximum inspiration as assessed by diaphragm ultrasound can rule out nighttime hypercapnia in patients with slowly progressive myopathies and may be used if weakness of mouth closure precludes reliable measurement of FVC or MIP. This finding corresponds with a previous study that showed that diaphragm mobility on ultrasound is related to FVC and MIP in healthy adults (13). The same study also revealed a significant (but slightly weaker) association between spirometric measures and the increase in diaphragm thickness during inspiration. Of note, diaphragm thickening ratio was not predictive of nocturnal hypercapnia in the present work. This observation may be explicable by two reasons: First, in patients with genetic myopathies, diaphragm atrophy is likely to be present and possibly limits the muscle's ability to increase its thickness on contraction. Second, ultrasound assessment of diaphragm thickening only gives a two-dimensional perspective on diaphragm action, whereas inspiratory effort results from a three-dimensional displacement of the muscle, which may be better reflected by excursion velocity. Accordingly, it has been shown that the extent of diaphragmatic thickening for a given level of inspiratory effort varies considerably between participants and measurements (28). In fact, diaphragm thickening explains only one-third or less of the variability in inspiratory effort (28). In contrast, sniff velocity has been shown to correlate with invasively obtained inspiratory muscle strength (13), which likely explains why it proved to be more suitable in the present study. However, it was not possible to define a cutoff value below which nocturnal hypercapnia can be expected. Furthermore, this study suggests that lung function tests might be more sensitive in predicting nocturnal hypoventilation than diaphragm ultrasound. Both observations may be ascribed to the small sample size and reflect that, regarding the use of diaphragm ultrasound in patients with slowly progressive neuromuscular disorders, this study has to be considered as preliminary.

It may be considered a weakness of this study that nighttime NIV had already been established in 16 patients. Regular use of NIV during sleep may enhance diaphragm strength and endurance during the day, but specific effects are unknown and have not been studied in patients with neuromuscular disease. Daytime tests of respiratory muscle performance might have been worse if NIV had not been used for a longer period of time or never before in these patients. However, it can be assumed that in this case, test accuracy of the parameters tested here would probably have been even better than reported.

## Conclusion

In slowly progressive myopathies, nocturnal hypercapnia is predicted by FVC <60% of the predicted value or by MIP <120% of the LLN. Furthermore, it can be excluded with clinically acceptable sensitivity by means of diaphragm excursion velocity on ultrasound during a voluntary sniff maneuver. All three measures allow for preselection of patients at risk for sleep-related hypoventilation and may steer the clinical decision when to proceed to sleep studies and overnight capnometry.

## Data Availability Statement

The raw data supporting the conclusions of this article will be made available by the authors, without undue reservation.

## Ethics Statement

The studies involving human participants were reviewed and approved by Ethikkommission der Ärztekammer Westfalen-Lippe und der WWU Münster, Reference Number: AZ 2016-072-f-S. The patients/participants provided their written informed consent to participate in this study.

## Author Contributions

MB, JS, WR, and SH planned the study. JS, RL, and CH were responsible for data collection. PY and SH helped with the recruitment of patients. Statistical analyses were performed by DG, JS, and MB. JS, RL, and MB wrote the manuscript, which was critically revised by H-JK, WR, and MD. All authors contributed to the article and approved the submitted version.

## Funding

This study was supported by Sanofi-Genzyme, Neu-Isenburg, Germany. The funders had no role in study design, data collection and analysis, preparation of the manuscript, or the submission process.

## Conflict of Interest

JS has been supported by the Else-Kröner-Fresenius Stiftung (Grant SP A109) and by the Kommission für Innovative Medizinische Forschung an der Medizinischen Fakultät Muenster (IMF Grant SP 11 18 15), Deutsche-Herzstiftung (DHS Grant SP 01/09), Scuola Superiore Sant‘Anna Pisa, Italy (Ph.D. Programme Translational Medicine), and Chiesi and Boehringer Ingelheim outside this work. MB has been supported by Loewenstein Medical outside this work. PY and MB have received speaker honoraria and travel grants from Sanofi Genzyme, Sanofi-Aventis, UCB, and Loewenstein Medical. WR has received travel grants and speaker honoraria from Loewenstein Medical, Philips Respironics, Novartis, Inspire, and Boehringer Ingelheim. H-JK was supported by Deutsche Forschungsgemeinschaft (DFG) outside this work and received travel grants and/or speaking fees from Actelion, Bayer, GlaxoSmithKline, MSD Sharp & Dohme, and Pfizer Deutschland. MD reports to have received travel grants and/or speaking fees and/or fees for consulting from Actelion, Astra Zeneca, Bayer, Berlin Chemie, Boehringer, Chiesi, Hamilton, Loewenstein Medical, Intermune, Linde, Novartis, Pfizer, Philips Respironics, ResMed, Roche and Weinmann. The remaining authors declare that the research was conducted in the absence of any commercial or financial relationships that could be construed as a potential conflict of interest.

## Publisher's Note

All claims expressed in this article are solely those of the authors and do not necessarily represent those of their affiliated organizations, or those of the publisher, the editors and the reviewers. Any product that may be evaluated in this article, or claim that may be made by its manufacturer, is not guaranteed or endorsed by the publisher.

## Abbreviations

AHI: apnea hypopnea index
AUC: area under the curve
BMI: body mass index
DMD: Duchenne's muscular dystrophy
DM1: myotonic dystrophy type 1
DM2: myotonic dystrophy type 2
FEV1: forced expiratory volume in 1 s
FRC: functional residual capacity
FSHD: facioscapulohumeral dystrophy
FVC: forced vital capacity
IVC: inspiratory vital capacity
LLN: lower limit of normal
MEP: maximum expiratory pressure
MIP: maximum inspiratory pressure
NIV: non-invasive ventilation
PCF: peak cough flow
pCO_2_: partial pressure of carbon dioxide
p_tc_CO_2_: transcutaneous carbon dioxide tension
ROC: receiver operating characteristics
SpO_2_: peripheral oxygen saturation
TLC: total lung capacity.
